# Optical Fiber Temperature Sensors and Their Biomedical Applications

**DOI:** 10.3390/s20072113

**Published:** 2020-04-09

**Authors:** Paulo Roriz, Susana Silva, Orlando Frazão, Susana Novais

**Affiliations:** 1 CIDESD (ISMAI), N2i (IPMAIA), LABIOMEP (Porto Biomechanics Laboratory), 447-690 Maia, Portugal; paulororiz@ismai.pt; 2 INESC TEC—Institute for Systems and Computer Engineering, Technology and Science, Rua do Campo Alegre 687, 4169-007 Porto, Portugal; susana.o.silva@inesctec.pt (S.S.); susana.novais@inesctec.pt (S.N.); 3 Department of Physics and Astronomy, Faculty of Sciences of University of Porto, Rua do Campo Alegre 687, 4169-007 Porto, Portugal

**Keywords:** optical fiber, temperature sensors, biomedical applications, healthcare

## Abstract

The use of sensors in the real world is on the rise, providing information on medical diagnostics for healthcare and improving quality of life. Optical fiber sensors, as a result of their unique properties (small dimensions, capability of multiplexing, chemical inertness, and immunity to electromagnetic fields) have found wide applications, ranging from structural health monitoring to biomedical and point-of-care instrumentation. Furthermore, these sensors usually have good linearity, rapid response for real-time monitoring, and high sensitivity to external perturbations. Optical fiber sensors, thus, present several features that make them extremely attractive for a wide variety of applications, especially biomedical applications. This paper reviews achievements in the area of temperature optical fiber sensors, different configurations of the sensors reported over the last five years, and application of this technology in biomedical applications.

## 1. Introduction 

Requests for detection of environmental changes through physical, chemical, or biological parameters have grown rapidly over the last decades. There is great interest, not only in the scientific community, but also in the industry, in developing new sensing devices based on optical fibers, in an effort to exploit their intrinsic characteristics and find new application fields. In order to compete with conventional sensors, these sensors need to be trustworthy, robust, highly sensitive, and affordable.

Various techniques are being developed in response to the increased need for non-destructive techniques that can monitor environments that are difficult to access; some of the most promising ones are based on optical fiber sensors (OFSs). The ability to have small devices physically near the objects or media being sensed brings about new opportunities; for example, in structural monitoring and industrial or pharmaceutical applications [1]. Considering their distinct working principles, there are several types of OFSs, which normally are separated into two classes: (i) extrinsic, where the optical fiber is only a medium to convey light to and from a separate element or space, and (ii) intrinsic, where the optical fiber constitutes the sensing element [2]. 

The advantages of using OFSs are well known—small dimensions, capability of multiplexing, chemical inertness, and immunity to electromagnetic fields. Furthermore, these sensors usually present good linearity, rapid response for real-time monitoring, and high sensitivity. Thus, OFSs present several features that make them highly sensitive to external perturbations and allow them to be embedded in materials [3], which is attractive in a wide variety of applications, namely in the medical, aerospace, and wind energy industries. They have also been applied in the oil and gas industry, taking advantage of distributed sensing capability, and in several fields, such as electrical engineering, materials science, biology, chemistry, physics, and optics [4]. There is no doubt that depending on the application and parameter or configuration for which the sensors are developed, the possibilities are huge [5,6]. Currently, the fiber sensing field is immense, and there is a wide variety of methods to classify the sensors, according to:

◦ The application: temperature, strain, displacement, current, magnetic fields, pressure, torsion, bending, vibration, humidity, lateral load, refractive index, detection of bio-molecules or chemical species [4].
◦ The measurable spatial scope: point sensors, quasi-distributed sensors, and fully distributed sensors [6].
◦ The modulation process: intensity, phase, state of polarization, and wavelength shift (frequency) [4].
◦ The working principle: optical fiber gratings (fiber Bragg grating (FBG), chirped fiber Bragg grating, tilted fiber Bragg grating (TFBG) and long period grating), interferometry (Fabry-Pérot (FP), Mach-Zehnder, Michelson, Sagnac, high birefringence fiber loop mirror sensors, and multimode interferometer) [7,8,9,10], distributed sensors (Raman scattering, Rayleigh scattering and Brillouin scattering) [8], or polarization-optical time domain reflectometry sensors [11,12].

The characteristics mentioned earlier make temperature monitoring using optical fibers very interesting. Diverse temperature sensors based on optical fibers have been projected over the last few years [13,14,15]. These optical sensing platforms are aligned to different techniques, such as FBG [16,17], Surface Plasmon Resonance (SPR) [18,19], side-polished fibers [20,21], photonic crystal fibers (PCF) [22,23,24], fiber FP interferometers [25,26,27,28,29], and tapered fibers [30,31]. Of the two types of fibers—glass and plastic optical fiber (POF) [32,33] is considered particularly advantageous owing to its excellent flexibility, easy handling, great numerical aperture, large diameter, and the fact that plastic is able to resist smaller bend radii more than glass [33,34].

A few of these technologies will be addressed specifically.

### 1.1. Fiber Bragg Grating 

In 1978, Kenneth O. Hill reported the first work on FBGs and their applications, both in optical communications and optical sensor systems [35]. Since then, this type of sensors has been widely applied in the measurement of different parameters, such as physical, chemical, clinical, biomedical and electrical parameters in the energy, aerospace and civil fields. They are simple, intrinsic sensing elements, which can be photo-inscribed into silica fiber and offer all the advantages associated with fiber optic sensors. Typically, a FBG sensor can be seen as a selective photo-induced modulation of the optical fiber core refractive index. The FBG resonant wavelength (Bragg wavelength), λ*_B_*, is related to the effective refractive index of the core mode (*n_eff_*) and the grating period (Λ), according to Equation (1) [36]:

(1) $$\lambda_{B}=2n_{eff}\Lambda$$

**Figure 1:**
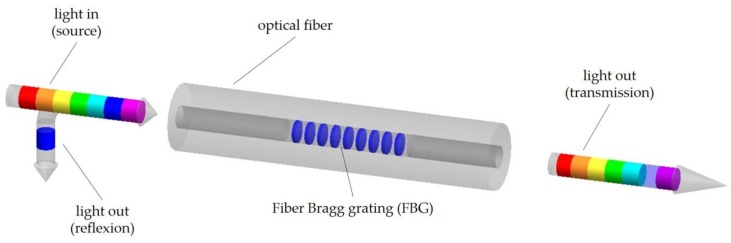
Schematic of a Bragg grating structure.

When the grating is illuminated by a broadband optical source, the reflected spectrum presents a sharp peak, which is caused by interference of light with the planes of the grating. Any perturbation on the grating (e.g., external strain or temperature variation) results in a shift in the Bragg wavelength, which can be detected either in the reflected or transmitted spectra [6]. See Figure 1.

### 1.2. Fabry-Pérot Interferometers

Interferometric optical fiber sensors are based on the principle of optical interference for the measurement of chemical or physical properties. These sensors can be an excellent solution for sensing because they can exhibit high sensitivity compared to FBGs, a wide dynamic range, multiplexing capacity, and low losses [37]. 

**Figure 2:**
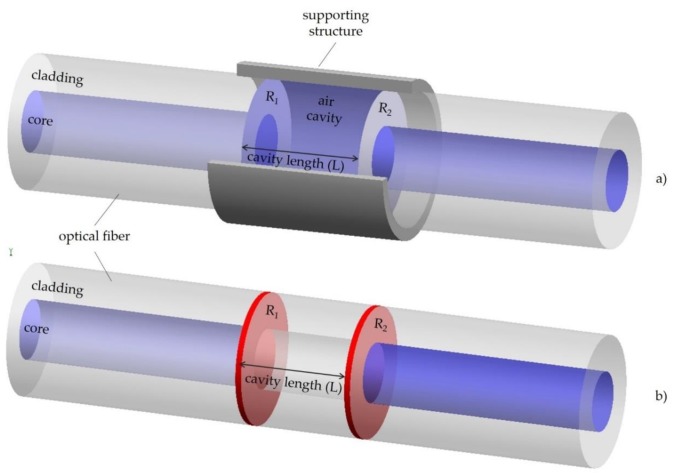
(**a**) Extrinsic and (**b**) intrinsic Fabry-Perot interferometer sensor, with reflectance *R_1_* and *R_2_*, separated by a determined distance, *L.* The supporting structure in (**a**) was partially removed.

One of the first works published on a fiber optic sensor based on FP interferometry was in 1982 by Yoshino [38]. Since then, rapid evolution has occurred in the field. FP interferometers are normally constituted of two parallel reflecting surfaces, with reflectance *R_1_* and *R_2_*, separated by a determined distance, *L*, as exemplified in Figure 2 [39]. The FP interferometer can be developed by intentionally building up reflectors externally to the fibers (Figure 2a), or internally (Figure 2b), being classified into two categories: extrinsic and intrinsic sensors [40,41].

In the case of extrinsic sensors, air cavity can be formed through a supporting structure, such as the one shown in Figure 2a. These sensors are advantageous for obtaining high finesse interference signals [42], bearing in mind that high reflecting mirrors may be used, although the manufacturing process is simple and no expensive equipment is needed. However, they have reduced coupling efficiency, and careful and precise alignment is required [43]. 

The intrinsic FP interferometer fiber sensor has reflecting components within the fiber itself. There are several ways to form this type of sensor, such as micro machining [44,45,46,47], by using two FBGs in series [48,49], through chemical etching [50,51,52], by thin film deposition [53,54,55], using special fibers [56,57], or even creating an air bubble in fibers [58,59]. In the simplest form, when the cavity has low-reflectivity mirrors, it can be approximated to a two-wave interferometer. In such cases, the reflection spectrum is essentially determined by the phase difference, *δ_FP_*, between the waves generated in the two reflections [59], which is described by:

(2) $$\delta_{FP}=\frac{4\pi nL_{FP}}{\lambda }$$

where, *n*, *L_FP_*, and *λ* are the effective refractive index of the cavity material, the physical length of the cavity, and the wavelength of incident light, respectively. When there is an external perturbation, such as variation of strain, temperature, or other parameters detectable by the sensors, both the cavity length and effective refractive index are able to change, translating into a shift of phase difference. This shift can be easily monitored by using a suitable interrogation system [39,59]. Table 1 presents the main characteristics of the different intrinsic FP sensors used for temperature measurements reported over the last five years in the literature. 

### 1.3. Multimode Fiber Interferometers Sensors

**Figure 3:**
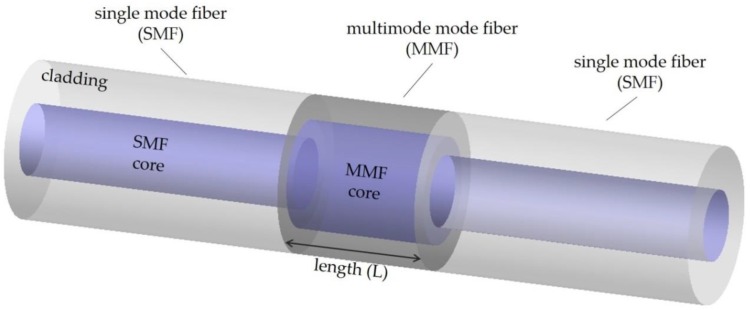
Schematic configuration of the single-mode-multimode-single-mode fiber structure.

A scheme of a typical multimode fiber interferometer (MMI) sensor is presented in Figure 3, where a section of the multimode fiber (MMF) is sandwiched between two single-mode fibers (SMFs) [78]. This is the so-called single-mode-multimode-single mode (SMS) fiber structure; however, MMIs can also be obtained using a single-mode-multimode fiber configuration [79]. It has a series of advantages that allows it to be used as a sensor—simple structure, low cost, small size, and high stability. Some of the parameters that have been monitored with this kind of sensor are strain and temperature [80], displacement [81], refractive index [82], and microbend [83].

The subjacent operating principle of this kind of sensor is the MMI excitation between modes in the MMF section, which can be influenced by external perturbations [84,85], i.e., the fundamental mode that propagates along the SMF will couple into the MMF, exciting many modes, each of which has a different propagation constant [6]. After passing through the multimode section, they reconnect to the SMF. Since each mode has already experienced a different phase shift, the modes interfere.

The main configurations of the MMI structures are simple and enable the detection of a change in the refractive index of the surrounding medium, i.e., due to the high interaction of the evanescent field with the external environment. The manufacturing of new concepts of MMIs can be greatly enhanced through the combination of fiber optics with nano-structure technology and the use of sensitive thin films [86,87,88,89]. Sensors based on MMI, allied with the functionalization with thin films as sensitive elements, could open new fields for optical fiber sensor applications. Functional materials can be deposited on the side- or end-face of fibers with different techniques, such as spin-coating, dip-coating, thermal evaporation, or sputtering [90]. The use of polymeric sensitive materials in optical fiber sensors has the advantage of enhanced response time with better sensitivity and selectivity [6,91]. Table 2 reports the main configurations for temperature parameter using MMI sensors reported over the last five years. 

## 2. Biomedical Optical Fiber Temperature Sensors

In clinical practice, patient temperature is a basic diagnostic procedure and often a critical control parameter, as in hyperthermia therapy [106]. Almost all chemical processes and reactions are temperature dependent, justifying temperature sensors as the largest class of commercially available OFS. Nevertheless, they are quite few compared to the large number of schemes that have been proposed but never reached commercialization [107]. Thermocouple and thermistor devices have been extensively used for temperature measurements in clinical practice. However, due to the presence of metallic conductors, they are inappropriate for clinical procedures involving incident radio frequency (RF), EM or microwave (MW) fields [108,109,110]. To overcome these limitations, fiber optic fluorescent techniques have been proposed. The fluoroptic technology uses fluorescent materials, such as rare-earth phosphors or gallium arsenide (GaAs), and an adequate light source to excite them. Temperature can be determined by measuring fluorescence emission decay times in the fluoroptic probes [111,112,113,114]. Solid state materials can also be used for fluorescence thermometry and some schemes have been presented for biomedical purposes, using the ruby [111,115] and the trivalent-chromium ion doped material [116]. An excellent review of fluorescent intensity, the first technique being proposed, and fluorescence lifetime based systems was published by Grattan and Zhang [107]. 

The Luxtron m3300 is a currently available fluoroptic system that can be used in biomechanical and biomedical laboratory settings (LumaSense Technologies, Santa Clara, CA, USA). Its non-metallic probe has a phosphorescent sensor localized at the probe tip and can provide real-time temperature measurements, ranging from 0 °C to 120 °C, with an accuracy of ±0.2 °C and 2 °C, respectively [117]. The probe has a 0.5 mm outer diameter (OD) and is protected with a Tefzel^®^ ethylene-tetrafluoroethylene (ETFE) fluoropolymer jacket allowing its use in magnetic resonance imaging (MRI), radio frequency (RF), or microwave (MW) environments and during ablation procedures [118,119]. A reported limitation of the Luxtron fluoroptic probe is its propensity to record higher temperatures compared to reference thermocouples sensors [120]. This was observed under localized heating at distances less than 4 mm from the laser source [120]. The T1™ Fiber Optic Temperature Sensor (Neoptix, Inc., Québec, QC, Canada) is also a commercially available OFS based on a GaAs semiconductor crystal located in the tip of the sensor. Sensor specifications include a temperature range from −272 °C to +250 °C, an accuracy of ±0.2 °C, a resolution of 0.1 °C, and a response time of 500 ms [121]. The outer protective jacket is made of polytetrafluoroethylene (PTFE) Teflon™ with 1.15 mm OD. It has been used to monitor temperature during cryogenic [122] and laser ablation procedures [123,124] as well as in non-incineration methods for sterilizing hospital infectious wastes [125]. Unfortunately, fluorescent materials are relatively bulky and expensive, which increases the cost of these systems [109]. 

**Figure 4:**
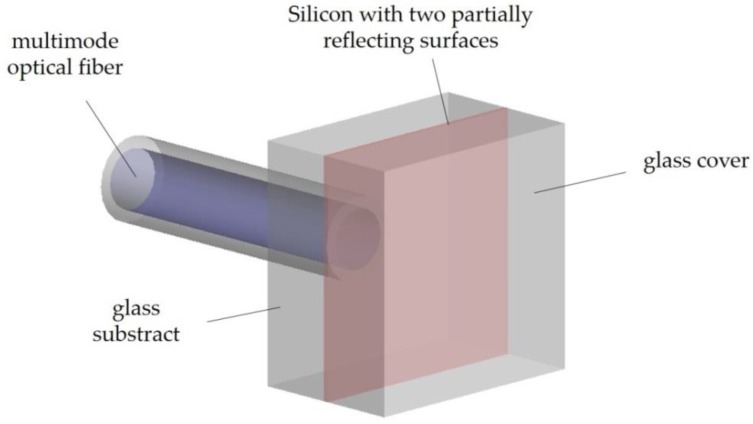
Schematic drawing of the temperature sensor proposed by Wolthuis et al. (Adapted from [109].)

Interferometric technology was explored by Wolthuis et al. [109], who presented a FP temperature sensor based on a LED-microshift method (Figure 4). It consisted of a light emitting diode (LED) light source, used to interrogate changes in optical cavity depth occurring between two reflectance peaks, and of a dichroic ratio technique used to analyze the returned signal [126]. The authors argued that the method was more sophisticated than others involving FP sensors, such as incremental, intensity, white-light, and LED-deep cavity. The optical cavity consisted of a thin layer of silicon packed between two pieces of glass. Temperature variations cause the silicon refractive index to change and, consequently, the light being reflected. Sensor performance fulfilled (American Association for Medical Instrumentation) AAMI specifications presenting a span linearity of 1% and sensitivity of 0.1% ratio change per °C. Temperature resolution and accuracy were 0.2 °C (0.02 °C with averaging) and 0.1 °C, respectively, for a measurement range from ~15 to ~55 °C. The sensor was able to reach 90% of its final value for a temperature change from ice to boiling water in about 200 ms [109]. RJC Enterprises, LLC (Bothell, WA, USA) is commercializing this type of sensor with some possibilities of customization (e.g., total assembly length and capillary pedestal length). 

**Figure 5:**
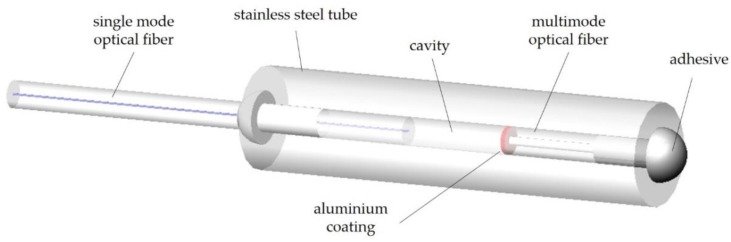
Schematic drawing of the temperature sensor proposed by Rao and Jackson. A stainless steel tube was made transparent to allow component visualization. (Adapted from [127].)

An interferometric configuration was also applied by Rao and Jackson [127] to propose a high-resolution temperature sensor (Figure 5). It consisted of a miniature extrinsic fiber optic-based Fizeau temperature sensor, with a cavity length of several hundred microns and a dual-wavelength pseudo-heterodyne phase detection scheme. A measurement resolution of 0.006 °C, a 1% span linearity over a temperature range of 27.3 to 62.5 °C, and a bandwidth of 30 Hz were achieved. To get temperature independent measurements, two FBG sensors located in a bimetallic beam were monitored interferometrically. Sensor performance meets or exceeds medical requirements but, to our best knowledge, it is not being marketed.

**Figure 6:**
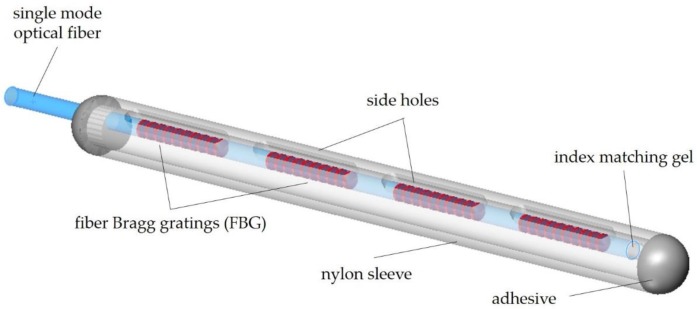
Schematic drawing of the temperature sensor proposed by Rao et al. (Adapted from [108].) A nylon sleeve was made transparent to allow component visualization.

Previously mentioned sensors are point sensors, i.e., they provide information only at the site they are placed and may be insufficient for a more complete clinical assessment. Multiplexing techniques using FBG sensors can contribute to overcoming this spatial constraint. The first configurations for medical use were proposed by Rao et al. [108] and Rao [128], consisting of an array of four in-line FBG (4 mm length each and 10 mm spaced) and a simple monochromator for demultiplexing the wavelength encoded signals (Figure 6). Wavelength-shifts induced by temperature variations were measured using a high-resolution drift-compensated interferometric detection scheme, based on a bulk unbalanced Michelson interferometer. To minimize strain effects, the probe end was sealed with a nylon sleeve of 1 mm OD. A resolution of 0.1 °C and an accuracy of ±0.2 °C, over a temperature range of 30 °C to 60 °C, were achieved in bench tests [129].

The above sensor was proposed for in vivo temperature monitoring during tumor therapy; in vivo trials occurred later using a similar configuration that was proposed by the same research group (Applied Optics Group, The University Canterbury, Kent, UK) [130]. A portable sensing unit with five in line FBGs was used. The source was a super luminescent diode (SLD) and the detector a miniature charge-coupled device (CCD) based spectrometer. Sensor resolution was 0.2 °C. This type of sensor was used to monitor hyperthermia treatments of the kidney and liver of rabbits [130,131]. Nevertheless, it was not applied in clinical settings because a nonlinear response of some FBG sensors and an initial system calibration drift exceeding 10 °C was reported [132]. To overcome these limitations, a polymer coated FBG (PFBG) probe was proposed [132]. It consisted of a 0.5 mm OD prototype with 10 FBG sensors at 5 mm intervals and 50 mm length. The PFBG sensor closely followed the behavior of well-established commercial hyperthermia thermometry probes. A swept wavelength laser-based readout system was capable to achieve 0.1 °C precision, while maintaining a better than 0.5 °C stability over 10 h and an absolute measurement accuracy of ±0.25 °C [132]. The sensor was tested only under simulated MW hyperthermia treatment to a tissue equivalent phantom.

The potentialities of other coating materials were explored in both MRI environments and cryoablation procedures. Samset et al. [133] were able to observe the dynamics of the freezing process during in vivo cryoablation of a porcine liver in a MRI room. Two multiplexed FBG array probes were used—one coated with polyimide (1.25 OD), the other with titanium (1.40 mm OD). The materials were considered biocompatible, sterilizable, and immune to EM interference. The probes exhibited excellent mechanical stability under cooling (−195.8 °C), hitting over a sharp edge, and bending to a radius of 20 mm at body temperature. The sensor, with 10 in-line FBGs, was calibrated for temperature through immersion in liquid nitrogen (−195.8 °C), ice slush (0 °C), and boiling water (100 °C). A reference platinum thermos resistance (Pt-100) was used to obtain the wavelength to temperature conversion parameters. Temperature measurements performed during prostate cancer cryosurgery confirmed FBG sensor thermometry potentialities for clinical applications [134,135]. A commercial reusable multiplexed FBG temperature monitor system was used (TMS, MultitempTM 1601, InvivoSense, Trondheim, Norway). Ultrafine 17 gauge needles were used to guide the sensor to the target tissue and temperatures were measured in four and eight FBG sensors with 10 mm and 5 mm distance intervals, respectively. Temperatures of about −40 °C or −60 °C were attained during the cryosurgery treatments, which are in the range (−100 °C and +130 °C) of these FBG multiplexed sensors.

The use of FBG arrays or other spatially distributed sensing techniques (e.g., modal modulation techniques) is also emerging for healthcare applications, namely for distributed body temperature monitoring using FBGs arrays. Martin et al. [136] proposed the use of FBG sensors to measure and monitor patient body temperature non-intrusively on a smart bedsheet. The use of FBG sensors allows a smart bedsheet to have the look and feel of a conventional bedsheet since FBG sensors have a very thin and light linear geometry. Additionally, they are dielectric in nature and have total immunity to electromagnetic and radio frequency (RF) interferences.

FBG sensors also prove to be useful in the field of prosthesis design and testing, namely, to measure polymerization temperature profiles of cemented hip mantles [137]. Peak temperatures of 110 °C reached within 300 s and stabilized to room temperature after 3600 s were measured with a resolution of 1 με and precision of ±5 με.

**Figure 7:**
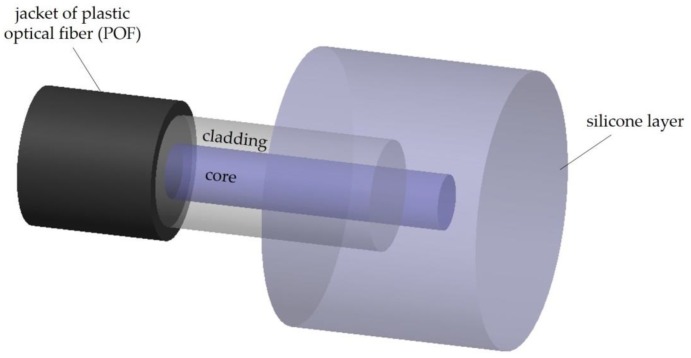
Schematic drawing of the temperature sensor proposed by Cennamo et al. (Adapted from [138].)

Cennamo et al. [138] presented a novel optical temperature sensor in a multimode plastic optical fiber with a polymethyl-methacrylate (PMMA) core of 980 μm, having a silicone layer (fluorinated polymer cladding of a 20 μm) deposited around the fiber tip for hyperthermia in cancer treatment (the optical fiber sensor is especially suited for minimally invasive measurement of local tissue temperature). This approach is dedicated to a portable temperature-sensing platform centered on low-cost, small size, and easy-to-use configuration (Figure 7). In this configuration, the silicone layer is extremely vital to monitor the temperature of the medium, since without it, the sensing structure is sensitive to the refractive index of the medium itself. Besides, in the hyperthermia treatment, the biocompatible silicone layer also contributes to minimally invasive measurement of local tissue temperature. A simple experimental setup, based on a halogen lamp as light source and two spectrum analyzers (one for the signal and the other for the reference) is used to monitor the sensor’s response at different wavelengths (652 nm and 736 nm). The proposed sensor behaves differently when the wavelength changes. The fiber temperature sensor proposed could be used to monitor temperatures in hyperthermia treatment, in the desired 35 to 45 °C range, with a resolution of about 0.1 °C. 

Recently, the use of hyperthermia—a technique that exploits heat to alter the biological state of tumors—as adjuvant therapy has been shown to increase the efficiency of the treatment. Through controlled elevation of tumor temperature, tumor physiology, including oxygenation and blood flow, can be altered. This control of the tumor micro-environment, allows the clinician to better plan and carry out the therapy.

It is known that the temperature is one of the vital signs and a crucial and routinely monitored parameter in medicine, which is measured using a variety of technologies [139] in all clinical settings, including surgeries, oncology treatment, and intensive care units [140]. In healthcare, the temperature sensing requirements are application dependent, but generally, a temperature range of 35–45 °C with a resolution of at least 0.1 °C is required [141]. 

The required response time of the temperature sensor is also application dependent [142]. For some thermal treatment procedures, such as high-intensity focused ultrasound ablation, the coagulative temperature (43.5–57.0 °C) is reached in less than 30 s [143], while for laser ablation, this can lie between 5 and 15 min [143].

**Figure 8:**
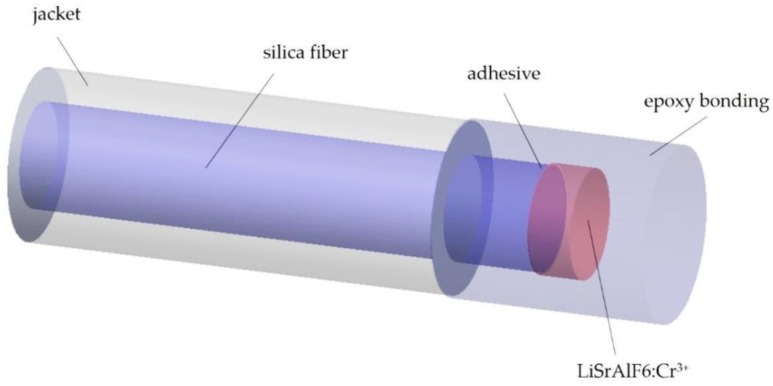
The probe of fiber temperature (Adapted from [145].)

Wu et al. [144] proposed a fluorescence optic-fiber sensor particularly adapted to the field of biomedicals in the range of 20 °C to 50 °C, based on modulated phase-locked detection (PLD) with pulse modulation signal references (PMSR). The probe of the detection system is composed of an optical system used to excite and transmit fluorescence and an electronic system used to probe and process the fluorescence signals. This probe is placed in a pure silicone catheter, which is to be planted at the location of the prostate. To monitor the temperature of the tissue under radiofrequency treatment, the temperature probe is placed in the catheter along with the radiofrequency treatment. The temperature probe is depicted in Figure 8. The volume of the LiSrAlF_6_: Cr^3+^ sample used was circa 0.3 × 0.2 × 0.3 mm^3^; nevertheless, this specific size is not critical. 

**Figure 9:**
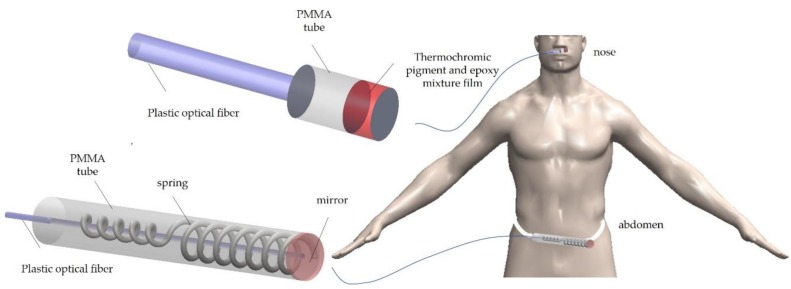
Schematic drawing of the structures of the nasal-cavity and abdomen-attached fiber-optic sensors (Adapted from [146].)

Wook et al. [145] proposed two types of non-invasive optical fiber respiration sensors (Figure 9) that can measure respiratory signal during magnetic resonance image acquisition. These sensors were based on thermo-chromic material deposited onto the tip of plastic optical fiber for respiratory monitoring inside an MRI system. The reported sensors have two different applications; one of them was a nasal-cavity attached sensor that can measure the temperature variation of air-flow using a thermochromic pigment, and the other one, was an abdomen attached sensor that can measure abdominal circumference change using a sensing part composed of polymethyl-methacrylate tubes, a mirror, and a spring. They measured the modulated light guided towards the detectors in the MRI control room via optical fibers due to the respiratory movements of the patient in the MR room; the respiratory signals of the optical fiber respiration sensors are compared with those of the BIOPAC^®^ system. The authors verified that respiratory signals can be obtained without deteriorating the MR image. The intensity of the reflected light was changed by the variation of the distance between the mirror and the distal end of the plastic optical fiber according to abdominal movement.

Tosi et al. [146] proposed a FBG array-based sensing probe installed on a device for radiofrequency thermal ablation (RFTA). The probe was made of five FBGs with 0.5 cm active area and 1 cm spacing, to provide quasi-distributed thermal pattern measurements. Multiple experiments have been conducted on porcine liver, reporting a temperature pattern along the ablation axis. Thermal maps allowed quantifying of the exposure of each part of the tissue to the high temperature field and provided a comparison between different procedures. The achieved results showed the possibility of embedding FBG arrays on ablation devices in order to dynamically estimate the efficiency of the procedure and predict the ablation output. Such results have significant importance for hepatic tumors, in which the electrical properties of the liver limit the RFTA to tumors up to ~3 cm in size.

The same team [147], in a similar work, proposed a distributed temperature sensor (DTS) with a submillimeter spatial resolution for the monitoring of RFTA in porcine liver tissue. The DTS demodulates the chaotic Rayleigh backscattering pattern with an interferometric setup to obtain the real-time temperature distribution. A measurement chamber has been set up with the fiber crossing the tissue along different diameters. Several experiments have been carried out measuring the space-time evolution of temperature during RFTA. The work showed cases that had temperature monitoring in RFTA with unprecedented spatial resolution and that which can be exportable to in vivo measurements; the acquired data can be particularly useful for the validation of RFTA computational models.

Ding et al. [148] and Chen et al. [149] proposed one of the first applications of FBG for thermometry in laser ablation; they developed a distributed FBG sensor with length of 10 mm, encapsulated within a glass capillary, and used it to monitor temperature distribution in an ex vivo liver and an in vivo mouse. The authors assumed that uniform grating turns into chirped grating in a non-uniform temperature field. The algorithm implemented was useful to dynamically control the temperature of the target at 43 °C; the temperature at the edge and outside the target at 38 °C. FBGs also have been included in numerous studies involving temperature monitoring during cryoablation. The research team of Samset started working on the development of FBG sensors for temperature monitoring in tissues undergoing cryoablation, and afterwards used this sensor to calibrate MR thermometry [150]. The distributed sensor was an optical fiber (cladding diameter 125 µm) embedding 10 FBGs. The center-to-center separation between the sensing elements was 6.5 mm and thus the total length of the sensor array was 58.5 mm. Two arrays were fabricated and mounted inside polyimide and titanium tubes, both materials having magnetic susceptibility close to that of the tissue, with a total outer diameter of 1.4 mm. The sensor was calibrated in the range −189.5 °C to 100 °C. Mechanical stability and MRI compatibility were acceptable, allowing routine use.

Zou et al [151] proposed a miniature fiber optic temperature sensor based on the FP interferometric principle, which was specifically designed, fabricated, packaged, and tested for intravascular blood temperature measurements during thermal angioplasty. The FP fiber optic sensor was fabricated by using chemical etching and thermal deposition, and compared with other fiber optic temperature sensors. The FP fiber optic sensor was notable for its remote-sensing capability and high-spatial resolution point measurement. An in-vivo experiment was performed by using a swine model. During the animal test, intravascular blood temperature was obtained at different locations in the coronary artery to demonstrate the capability of the fiber optic sensor. In order to demonstrate the sensor’s usage in angioplasty applications, the rise and drop of local intravascular blood temperatures were successfully captured by the fiber optic sensor.

Najafi et al. [152] proposed and tested SmartSox, designed and made by Novinoor LLC (Wilmette, IL, USA), which is based on highly flexible fiber optics embedded in a comfortable standard sock, in patients with diabetic peripheral neuropathy. Using an optical amplifier and signal processing, SmartSox used five embedded highly flexible and thin (<0.3 mm) fiber optic sensors based on fiber Bragg gratings (FBGs) that were woven into a comfortable sock to measure plantar temperature and pressure under the first metatarsal head (MTH), fifth MTH, midfoot, and hind foot. This allows simultaneous measurements of temperature, plantar pressure, and toes range of motion, which makes it suitable for objectively assessing lower extremity regions at risk. The authors of this work found a moderate agreement in foot temperature changes between SmartSox and an infrared thermal camera [153].

Fook et al. [154] presented a novel approach of using fibers to provide the actual sensors itself used Internet of Things (IoT) applications developed to assist medical staff in caring for residents in nursing homes. Specially designed and packaged highly sensitive FBG-based optical fiber sensors are developed for use in a monitoring and alert system that oversees residents continuously without disturbing them, and automatically alerts medical staff during emergencies through mobile devices such as mobile phones or tablets [155]. The system is able to monitor temperature and detect the onset of high fever in residents. An IoT FBG-based sensor button was also developed to allow the residents to call or alert medical staff when necessary. The monitoring and alert system was primarily based on FBG technology. Using FBG technology, uniquely designed FBG sensors are packaged into IoT devices such as IoT sensor mat, IoT thermometer, and IoT button. They term the packaged Fiber-based IoT sensors as F-IoT devices because they used fibers to provide the actual sensors itself. In this kind of project, the sensitivity of the sensor mat can be adjusted based on sensor design, number of sensors, placement, and packaging material. A sleeve-based FBG design was selected by the authors due to its robustness and high sensitivity. Eight sensors were used and placed into two rows. The authors of this work took into account user comfort when designing the mat. The FBG sensor array was packaged onto a polycarbonate sheet. The final packaged IoT sensor mat was placed on top of mattresses to monitor vital parameters of residents in nursing homes.

## 3. Final Remarks

This paper reviewed achievements in the area of temperature optical fiber sensors, where different configurations of the sensors reported from 2015 to 2020 were presented and their possible potential for biomedical applications studied. 

In view of this review article, we can mention that the use of FBG sensors and spatially distributed sensing techniques is assuming high relevance for non-intrusive monitoring of temperature and other clinically relevant parameters, since they retain the mechanical stability of the optical fiber and also avoid referencing issues. 

Temperature is a vital sign and a crucial and habitually monitored parameter in medicine; it is measured using a variety of technologies in all clinical settings, including surgeries, oncology treatment, and intensive care units.

Thermocouple and thermistor devices are widely used for temperature measurements in clinical practice. However, due to the presence of metallic conductors, they are inappropriate for clinical procedures involving, for instance, incident radio frequency or microwave fields. Thus, it is important that new optical fiber technologies are developed, preferably portable temperature sensing platforms that offer low costs, small sizes, and easy-to-use configuration; and can use artificial intelligence for faster data analysis. 

The main advantages of optical fiber sensors for medical solutions are their small dimensions, chemical inertness, immunity to electromagnetic fields, rapid response for real-time monitoring, and ability to be embedded into materials.

## Figures and Tables

**Table 1:** Different configurations of Fabry-Perot sensors reported from 2015 to 2020.

Year	Configuration	Length (µm)	Range	Sensitivity	Ref
**2015**	SMF + dual HCF	33.84	20 to 60 °C	−0.4810 nm/°C	[60]
**2015**	Polymer capped on the end face of SMF	35.1	40 to 90 °C	0.249 nm/°C	[61]
**2015**	Rectangular air bubble between SMFs	~61	25 to 100 °C	2.0 pm/°C	[62]
**2015**	SMF + silicon pillar	200	20 to 100 °C	84.6 pm/°C	[63]
**2015**	MMF + Pyrex glass + silicon diaphragm	~32	−50 to 100 °C	6.07 nm/°C	[64]
**2016**	Air cavities with capillary fiber between 2 SMFs	~25-200	50 to 400 °C	0.8 pm/°C	[65]
**2016**	SMF + hollow-core photonic crystal fiber (PCF)	75	17 to 900 °C	0.94 pm/°C	[66]
**2016**	SMF + PCF	94	20 to 90 °C	9.17 pm/°C	[67]
**2017**	Etched MMF filled with UV adhesive	37.7	55 to 85°C	213 pm/°C	[68]
**2018**	SMF + Hollow core tube + SMF	~100	50–450 °C	0.902 pm/°C	[69]
**2018**	Fiber core near the end of a standard SMF	60	500 to 1000 °C	18.6 pm/°C	[70]
**2018**	SMF +capillary + nafion film	200	−30 to 85 °C	2.71 nm/°C	[71]
**2019**	SMF + HCF + HCF	210	30 to 200 °C	9.22 pm/°C	[72]
**2019**	SMF + HCF + grapefruit PCF	1229	25 to 70 °C	10.64 pm/°C	[73]
**2019**	SMF + HCF + long period fiber grating +SMF	474.4	31.5 to 82.4°C	135.19 pm/°C	[74]
**2020**	SMF + FBG + FBG + SMF	------	25 to 45 °C	307.6 pm/°C	[75]
**2020**	Parallel FPI	26 61	20 to 80 °C	0.74 pm/°C 1.37 pm/°C	[76]
**2020**	SMF + polarization maintaining PCF	150	300 to 800 °C	−92 pm/°C	[77]

**Table 2:** Multimode interference sensors reported in the literature from 2015 to 2020.

Year	Configuration	Length (mm)	Range	Sensitivity	Ref
**2015**	SMF + no core fiber (NCF) (diameter of 96 µm) + SMF	34.43	−30 to 100 °C	38.7 pm/°C	[92]
**2015**	SMF + offset SMF + SMF	46	30 to 270 °C	0.0449 nm/°C	[93]
**2015**	SMF + NCF + SMF	40	10 to 100 °C	5.15 nm/°C	[94]
**2017**	SMF + MMF (core of 105 µm) + SMF	44	15 to 75 °C	29.33 pm/°C	[95]
**2017**	SMF + polymer optical fiber (POF) + SMF	10	25 to 105 °C	102.2 pm/°C	[96]
**2017**	SMF + MMF + MMF + SMF	100	30 to 90 °C	6.8 pm/°C	[97]
**2018**	SMF + NCF (with alcohol solution within a silica capillary tube) + SMF	40	20 to 45 °C	0.49 dB/°C	[98]
**2018**	SMF + NCF	43.9	100 to 700 °C	6.8 pm/°C	[79]
**2018**	SMF + NCF	30	10 to 70 °C	13.6 pm/°C	[99]
**2019**	SMF + MMF + SMF	70	31.4 to 80.2 °C	21 pm/°C	[100]
**2019**	SMF + MMF + NCF + MMF + SMF	1	20 to 100 °C	33 pm/°C	[101]
**2019**	SMF + MMF + polarization maintaining fiber + MMF + SMF	32	20 to 40 °C	0.188 nm/°C	[102]
**2020**	SMF + NCF (with a gold film) + SMF	12	20 to 80 °C	37.9 pm/°C	[103]
**2020**	SMF + NCF (with coating) + SMF	15	−5 to 45 °C	−4.677 nm/°C	[104]
**2020**	SMF + hollow-core capillary waveguide + SMF	29.5	25 to 75 °C	−0.49 nm/°C	[105]
